# Gene model for the ortholog of *Roc1a* in *Drosophila persimilis*

**DOI:** 10.17912/micropub.biology.001043

**Published:** 2025-05-01

**Authors:** Megan E. Lawson, Kelsey Gammage, Calvin Dexel, Betty Duan, Elena M. Zerkin, Lindsey J. Long, Melinda A. Yang, Chinmay P. Rele, Laura K Reed

**Affiliations:** 1 The University of Alabama, Tuscaloosa, AL USA; 2 Oklahoma Christian University, Edmond, OK USA; 3 University of Richmond, Richmond, VA USA

## Abstract

Gene model for the ortholog of *Regulator of cullins 1a* ( *Roc1a* ) in the May 2011 (Broad dper_caf1/DperCAF1) Genome Assembly (GenBank Accession: GCA_000005195.1 ) of *Drosophila persimilis* . This ortholog was characterized as part of a developing dataset to study the evolution of the Insulin/insulin-like growth factor signaling pathway (IIS) across the genus *Drosophila* using the Genomics Education Partnership gene annotation protocol for Course-based Undergraduate Research Experiences.

**Figure 1. Genomic neighborhood and gene model for Roc1a in Drosophila persimilis:**
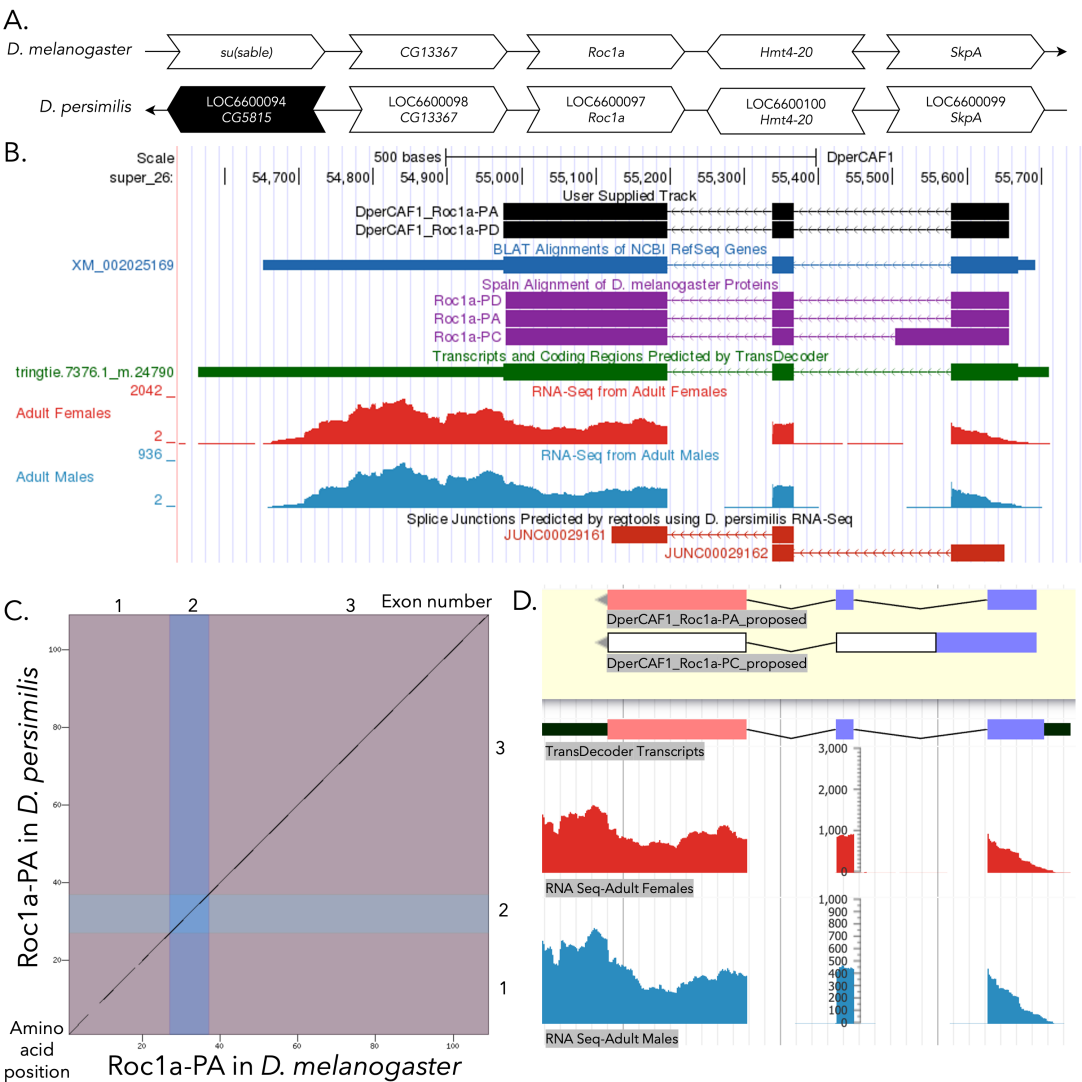
**
(A) Synteny comparison of the genomic neighborhoods for *Roc1a* in *Drosophila melanogaster* and *D.* *persimilis* .** Thin underlying arrows indicate the DNA strand within which the target gene– *Roc1a* –is located in *D. melanogaster* (top) and *D. persimilis* (bottom). The thin arrow pointing to the right indicates that *Roc1a* is on the positive (+) strand in *D. melanogaster* , and the thin arrow pointing to the left indicates that *Roc1a* is on the negative (-) strand in *D. persimilis* . The wide gene arrows pointing in the same direction as *Roc1a* are on the same strand relative to the thin underlying arrows, while wide gene arrows pointing in the opposite direction of *Roc1a* are on the opposite strand relative to the thin underlying arrows. White gene arrows in *D. persimilis* indicate orthology to the corresponding gene in *D. melanogaster* , while black gene arrows indicate non-orthology. Gene symbols given in the *D. persimilis* gene arrows indicate the orthologous gene in *D. melanogaster* , while the locus identifiers are specific to *D. persimilis* . **(B) Gene Model in GEP UCSC Track Data Hub** (Raney et al., 2014). The coding-regions of *Roc1a* in *D. persimilis* are displayed in the User Supplied Track (black); coding exons are depicted by thick rectangles and introns by thin lines with arrows indicating the direction of transcription. Subsequent evidence tracks include BLAT Alignments of NCBI RefSeq Genes (dark blue, alignment of Ref-Seq genes for *D. persimilis* ), Spaln of *D. melanogaster* Proteins (purple, alignment of Ref-Seq proteins from *D. melanogaster* ), Transcripts and Coding Regions Predicted by TransDecoder (dark green), RNA-Seq from Adult Females and Adult Males (red and light blue, respectively; alignment of Illumina RNA-Seq reads from *D. persimilis* ), and Splice Junctions Predicted by regtools using *D. persimilis* RNA-Seq ( PRJNA388952 ). Splice junctions shown have a minimum read-depth of 10 with >1000 supporting reads shown in red. **(C) Dot Plot of Roc1a-PA in *D. melanogaster* ( *x* -axis) vs. the orthologous peptide in *D. persimilis* ( *y* -axis).** Amino acid number is indicated along the left and bottom; coding-exon number is indicated along the top and right, and exons are also highlighted with alternating colors. **(D) Model of Roc1a-PA compared to Roc1a-PC in Apollo.** A screenshot of the Apollo instance housing the proposed CDS gene model of Roc1a-PA (identical to that of Roc1a-PD) and the likely absent Roc1a-PC isoform, containing in frame stop codons. The proposed models are shown at the top in the yellow region, while evidence tracks are found below in the white region. Exon reading frames are indicated in blue, green, and red, and implausible CDS sequence warnings (in this case, due to the in-frame stop codons) are indicated in white. Evidence tracks from top to bottom include Transcripts and Coding Regions Predicted by TransDecoder and RNA-Seq from Adult Females and Adult Males (red and light blue, respectively; alignment of Illumina RNA-Seq reads from *D. persimilis* ; PRJNA388952 ).

## Description

**Table:** 

*This article reports a predicted gene model generated by undergraduate work using a structured gene model annotation protocol defined by the Genomics Education Partnership (GEP; thegep.org) for Course-based Undergraduate Research Experience (CURE). The following information in this box may be repeated in other articles submitted by participants using the same GEP CURE protocol for annotating Drosophila species orthologs of Drosophila melanogaster genes in the insulin signaling pathway.* "In this GEP CURE protocol students use web-based tools to manually annotate genes in non-model *Drosophila* species based on orthology to genes in the well-annotated model organism fruitfly *Drosophila melanogaster* . The GEP uses web-based tools to allow undergraduates to participate in course-based research by generating manual annotations of genes in non-model species (Rele et al., 2023). Computational-based gene predictions in any organism are often improved by careful manual annotation and curation, allowing for more accurate analyses of gene and genome evolution (Mudge and Harrow 2016; Tello-Ruiz et al., 2019). These models of orthologous genes across species, such as the one presented here, then provide a reliable basis for further evolutionary genomic analyses when made available to the scientific community.” (Myers et al., 2024). “The particular gene ortholog described here was characterized as part of a developing dataset to study the evolution of the Insulin/insulin-like growth factor signaling pathway (IIS) across the genus *Drosophila* . The Insulin/insulin-like growth factor signaling pathway (IIS) is a highly conserved signaling pathway in animals and is central to mediating organismal responses to nutrients (Hietakangas and Cohen 2009; Grewal 2009).” (Myers et al., 2024). “ *Roc1a* (also known as *Rbx1* ) is a member of the SCF E3 ubiquitin ligase complex and was originally identified through sequence similarity with vertebrate and yeast homologs and biochemical interaction studies (Bocca et al., 2001). *Roc1a* deletion mutants are lethal between the first and second larval instars and *Roc1a* mutant clones in imaginal discs have cell proliferation defects (Noureddine et al., 2002). In addition, Roc1a and other members of the SCF E3 ubiquitin ligase complex function in the pruning of larval neurons by targeting the insulin-responsive kinase Akt for ubiquitination and degradation, thus inhibiting insulin signaling (Wong et al., 2013).” (Lawson et al., 2025). “ *D. persimilis* (NCBI:txid 7234) is part of the *pseudoobscura* species subgroup within the *obscura* species group in the subgenus *Sophophora* of the genus *Drosophila* (Sturtevant 1942; Buzzati-Traverso and Scossiroli 1955). It was first described by Dobzhansky and Epling (1944). The *pseudoobscura* species subgroup is endemic to the western hemisphere, where *D. persimilis* is found in the Pacific Northwest, sympatric with its sibling species *D. pseudooscura* (Markow and O'Grady 2005). Larvae of *D. persimilis* have been found feeding in sap fluxes from some oak species in California (Carson 1951), however its ecology is not fully elucidated (Powell 1997).” (Koehler et al., 2024).

We propose a gene model for the *D. persimilis* ortholog of the *D. melanogaster* *Regulator of cullins 1a* ( *Roc1a* ) gene. The genomic region of the ortholog corresponds to the uncharacterized protein LOC6600097 (RefSeq accession XP_002025205.2 ) in the May 2011 (Broad dper_caf1/DperCAF1) Genome Assembly of *D. persimilis* (GenBank Accession: GCA_000005195.1 ; *Drosophila* 12 Genomes Consortium et al., 2007). This model is based on RNA-Seq data from *D. persimilis* ( PRJNA388952 ; Yang et al., 2018 *)* and *Roc1a* in *D. melanogaster* using FlyBase release FB2023_02 ( GCA_000001215.4 ; Larkin et al., 2021; Gramates et al., 2022; Jenkins et al., 2022).

***Synteny***

The refrence gene, *Roc1a ,* occurs on chromosome X in *D. melanogaster* and is flanked upstream by *CG13367* and *suppressor of sable* ( *Su(sable)* ) and downstream by *Histone methyltransferase 4-20* ( *Hmt4-20* ) and *SKP1-related A* *( SkpA )* . The *tblastn* search of *D. melanogaster* Roc1a-PA (query) against the *D. persimilis* (GenBank Accession: GCA_000005195.1 ) Genome Assembly (database) placed the putative ortholog of *Roc1a* within scaffold super_26 ( CH479193.1 ) at locus LOC6600097 ( XP_002025205.2 )— with an E-value of 3e-48 and a percent identity of 74.53%. Furthermore, the putative ortholog is flanked upstream by LOC6600098 ( XP_002025206.2 ) and LOC6600094 ( XP_002025207.1 ), which correspond to *CG13367* and *CG5815* in *D. melanogaster* (E-value: 3e-129 and 3e-176; identity: 69.63% and 59.41%, respectively, as determined by *blastp* ) ( Figure 1A, Altschul et al., 1990). The putative ortholog of *Roc1a* is flanked downstream by LOC6600100 ( XP_026846708.1 ) and LOC6600099 ( XP_002025203.1 ), which correspond to *Hmt4-20* and *SkpA* in *D. melanogaster* (E-value: 5e-34 and 2e-117; identity: 52.03% and 98.77%, respectively, as determined by *blastp* ). The putative ortholog assignment for *Roc1a* in *D. persimilis* is supported by the following evidence: the synteny of the genomic neighborhood is almost entirely conserved, with only one upstream gene not matching, and all of the *BLAST* search results indicate that these ortholog matches are of very high-quality.

***Protein Model***

*Roc1a* in *D. persimilis* has one unique protein-coding isoform (Roc1a-PA and Roc1a-PD; Figure 1B ), encoded by mRNA isoforms *Roc1a-RA* and *Roc1a-RD* that differ in their UTRs, and contain three CDSs. Relative to the ortholog in *D. melanogaster* , the RNA CDS number is conserved for these isoforms. However, *D. melanogaster* also has a third isoform with two CDSs, Roc1a-RC, that is not present in *D. persimilis* (see: “special characteristics”). The sequence of Roc1a-PA in *D. persimilis* has 96.30% identity (E-value: 5e-76) with the protein-coding isoform Roc1a-PA in *D. melanogaster* , as determined by *blastp* ( Figure 1C ). Coordinates of this curated gene model (Roc1a-PD, Roc1a-PA) are stored by NCBI at GenBank/BankIt (accession **BK064560 , BK064561** , respectively **)** . These data are also archived in the CaltechDATA repository (see “Extended Data” section below).

***Special characteristics of the protein model***

mRNA isoform *Roc1a-RC* in *D. melanogaster* is similar to the other two isoforms, *Roc1a-RA* and *Roc1a-RD* . The difference in coding region between these isoforms is that *Roc1a-RA* and *Roc1a-RD* have three CDSs whereas *Roc1a-RC* has two CDSs, with its first CDS (FlyBase ID: 1_2094_0) spanning the length of the first two CDSs of *Roc1a-RA* and *Roc1a-RD* (FlyBase IDs: 2_2094_0 and 3_2094_0) combined (All CDS IDs based on FlyBase release FB2023_02; GCA_000001215.4 ; Larkin et al.,2021). In *D. melanogaster* , there are no in-frame stop codons for any of the isoforms, including in the longer first CDS of *Roc1a-RC* . However, in *D. persimilis,* there is an in-frame stop codon present in the first CDS of the potential Roc1a-PC isoform that prematurely stops translation of this isoform. This has been highlighted in Figure 1D by the white portion of the CDSs of Roc1a-RC indicating the presence of in-frame stop codons. This, in combination with the lack of RNA-seq data and TransDecoder Transcript predictions supporting the existence of Roc1a-PC, suggests that Roc1a-PC is likely absent from *D. persimilis* ( Figure 1D ) *.* This special characteristic of Roc1a-PC is similar to the observations described in *D. eugracilis* Lawson et al. (2025).

## Methods

Detailed methods including algorithms, database versions, and citations for the complete annotation process can be found in Rele et al. (2023). Briefly, students use the GEP instance of the UCSC Genome Browser v.435 (https://gander.wustl.edu; Kent WJ et al., 2002; Navarro Gonzalez et al., 2021) to examine the genomic neighborhood of their reference IIS gene in the *D. melanogaster* genome assembly (Aug. 2014; BDGP Release 6 + ISO1 MT/dm6). Students then retrieve the protein sequence for the *D. melanogaster* reference gene for a given isoform and run it using *tblastn* against their target *Drosophila* species genome assembly on the NCBI BLAST server (https://blast.ncbi.nlm.nih.gov/Blast.cgi; Altschul et al., 1990) to identify potential orthologs. To validate the potential ortholog, students compare the local genomic neighborhood of their potential ortholog with the genomic neighborhood of their reference gene in *D. melanogaster* . This local synteny analysis includes at minimum the two upstream and downstream genes relative to their putative ortholog. They also explore other sets of genomic evidence using multiple alignment tracks in the Genome Browser, including BLAT alignments of RefSeq Genes, Spaln alignment of *D. melanogaster* proteins, multiple gene prediction tracks (e.g., GeMoMa, Geneid, Augustus), and modENCODE RNA-Seq from the target species. Detailed explanation of how these lines of genomic evidenced are leveraged by students in gene model development are described in Rele et al. (2023). Genomic structure information (e.g., CDSs, intron-exon number and boundaries, number of isoforms) for the *D. melanogaster* reference gene is retrieved through the Gene Record Finder (https://gander.wustl.edu/~wilson/dmelgenerecord/index.html; Rele et al *.,* 2023). Approximate splice sites within the target gene are determined using *tblastn* using the CDSs from the *D. melanogaste* r reference gene. Coordinates of CDSs are then refined by examining aligned modENCODE RNA-Seq data, and by applying paradigms of molecular biology such as identifying canonical splice site sequences and ensuring the maintenance of an open reading frame across hypothesized splice sites. Students then confirm the biological validity of their target gene model using the Gene Model Checker (https://gander.wustl.edu/~wilson/dmelgenerecord/index.html; Rele et al., 2023), which compares the structure and translated sequence from their hypothesized target gene model against the *D. melanogaster* reference gene model. At least two independent models for a gene are generated by students under mentorship of their faculty course instructors. Those models are then reconciled by a third independent researcher mentored by the project leaders to produce the final model. Note: comparison of 5' and 3' UTR sequence information is not included in this GEP CURE protocol.

## Data Availability

Description: A GFF, FASTA, and PEP of the model. Resource Type: Model. DOI: https://doi.org/10.22002/ad6sn-vaq84
